# Considerations for trustworthy cross-border interoperability of digital identity systems in developing countries

**DOI:** 10.1007/s00146-024-02008-9

**Published:** 2024-08-07

**Authors:** Ayei Ibor, Mark Hooper, Carsten Maple, Jon Crowcroft, Gregory Epiphaniou

**Affiliations:** 1 Trustworthy Digital Infrastructure for Identity Systems, The Alan Turing Institute, London, UK; 2 WMG, University of Warwick, Coventry, UK; 3 Department of Computer Science, University of Calabar, Calabar, Nigeria

**Keywords:** Cross-border interoperability, Digital identity, Trustworthiness, e-Government

## Abstract

In developing nations, the implementation of Foundational Identity Systems (FIDS) has optimised service delivery and inclusive economic growth. Cross-border e-government will gain traction as developing countries increasingly look to identity federation and trustworthy interoperability through FIDS for the identification and authentication of identity holders. Despite this potential, the interoperability of FIDS in the African identity ecosystem has not been well-studied. Among the difficulties in this situation are the intricate internal political dynamics that have led to weak institutions, suggesting that FIDS could be used for political purposes; additionally, citizens’ or identity holders’ habitual low trust in the government raises concerns about data security and privacy protection. Similarly, vendor lock-in, cross-system compatibility, and ambiguous legislative rules for data exchange are other concerns. Interoperability is fundamentally necessary as a precondition for e-government services and serves as the foundation for the best possible service delivery in the areas of social security, education, and finance, as well as gender equality as demonstrated by the European Union (EU). Moreover, the integration of cross-border FIDS and an ecosystem of effective data governance will be created by unified data sharing via an interoperable identity system. Thus, in this study, we point to the challenges, opportunities, and requirements for cross-border interoperability in an African setting. Furthermore, we investigated current interoperability solutions such as the EU’s eIDAS and Estonian X-Road and proposed an approach for scoping requirements to achieve a fully functional interoperable identity ecosystem in the African setting. Our findings show that interoperability in the African identity ecosystem is essential for expanding the scope of e-government throughout the continent and for bolstering the smooth authentication and verification of identity holders for inclusive economic growth.

## Introduction

One of the Sustainable Development Goals (SDGs) of the United Nations (UN) is to provide legal identity for all citizens, which also includes birth registration by 2030 (World Bank Group 2018). Primarily, identification allows individuals to exercise their rights to be identified and legally recognised while the government and private sector depend on it for effective service delivery. In this sense, a national identity system is crucial to the identification and authentication of natural or legal persons and provides a means for citizens to access critical services including participation in formal political, social, and economic life (Gelb and Metz 2018). Achieving sustainable development is a key consideration of most national governments and forms the basis for inclusive and responsible identification schemes at the foundational level.

Fundamentally, national identification schemes are the pivot for social security, immigration, financial and economic inclusion, healthcare, voting, gender equality, transportation, and education (Colbern and Ramakrishnan 2020). Consequently, identity management plays a key role in promoting e-government by bringing services closer to the people. Modern identification systems are robust tools for the delivery of transparent administration, reduction in fraud and leakages for social benefits, providing adequate security for the citizens, extracting accurate biographic data for effective economic planning, and responding to natural disasters (Atick 2016). These seeming benefits are yet to solve the identification crisis in most developing nations as about 850 million people across the globe are yet to have access to a valid identity credential, mostly in sub-Saharan Africa and South Asia (The World Bank 2023a). According to the World Bank Identification for Development (ID4D) dataset (The World Bank 2023a), most people without official identification are residents of low-income countries. These sets of persons also include marginalised and vulnerable groups consisting of children, whose births were not documented in the civil registry of the affected country, women in rural areas with no access to digital services and adults below the age of 25 years.

Particularly, digital identification systems can improve how the public and commercial sectors provide services and lay the groundwork for new markets, services, and systems, such as e-government, cashless transactions, and the digital economy. Identification systems must, however, have high levels of coverage and inclusion within the population, and be resilient to fraud and error. Similarly, they must operate within a governance framework that protects personal data, fosters trust and accountability, and facilitates end-user control to fulfil their potential for sustainable development and increase public sector efficiency (African Union 2020; Bandura and Ramanujam 2021). There is a growing demand for digital identity to be mutually recognised and portable between countries in the modern digital age through an interoperability framework and in the context of regional and global integration and migration, which can be facilitated through trust and standards.

Interoperability, in this context, refers to the ability of disparate foundational identity systems to exchange data through seamless communication of identification and authentication information. These exchanges must be trustworthy or inherently secure, available, and reliable. Although there are complex internal political dynamics in most developing countries that result in weak institutions, and the lack of trust in government by the citizens (Grossman and Slough 2022; Hutchison and Johnson 2011), our findings show that interoperability is vital to widen the dimensions of e-Government. We also found that the notion of security and privacy protection concerns, legislation, cross-system compatibility, vendor lock-in, and unclear regulatory provisions for data sharing are other concerns that an interoperable identity ecosystem needs to address (Atick 2016; Stegemann and Gersch 2019). In this paper, the challenges, opportunities, and requirements for trustworthy cross-border interoperability are discussed. The structure of this paper is as follows; Sect. 2 provides the background and motivation of the research; Sect. 3 discusses the potential solutions of trustworthy interoperability in developing countries including their challenges and limitations; Sect. 4 presents the approaches towards trustworthy interoperability in an African setting; Sect. 5 presents the discussion of findings; Sect. 6 presents our concluding remarks.

## Background and motivation

The global economy requires the transnational exchange of data when authenticating ID holders or citizens who request services from government or private service providers. This data exchange must be based on openness and transparency, increased communication, collaboration, and sharing, which can be achieved through interoperability (Robles et al. 2019; Solvak et al. 2019; Sun et al. 2015). To create a global economy and e-government systems that allow ID holders to be seamlessly authenticated across borders, there is the need to ensure that identification data maintains its integrity, confidentiality, and privacy during the exchange of transactional data between stakeholders such as identity providers (IdP), service providers (SP), and relying parties (RP) (Fathalla et al. 2023; Guenduez et al. 2023).In the same sense, to enhance the trustworthiness of identification data, the presentation, validation, and verification of identity credentials must be lossless and bidirectional when authenticated across federating and interoperable ecosystems (Ibor et al. 2023; W3C Recommendation 2022). However, the implementation of cross-border interoperability including the actualisation of e-government services has been hampered by several factors. Some of these include the isolation and fragmentation of identity management systems (IdMS) in most developing countries, which discourages collaboration and creates redundancy of identification data across disparate isolated silos.

Essentially, identity management through the electronic identification (e-ID) of subjects plays a significant role in driving e-government services. This is because as technology evolves, accessing digital public services requires the use of electronic identification (e-ID) technology. Although there are concerns about the impact of digital services on ethnic minorities and vulnerable groups of people (Kemppainen et al. 2023a, b), who may find it difficult to acquire an e-ID, achieving an interoperable identity ecosystem either through identity federation or the use of decentralised identity ecosystems depends largely on the success of the e-ID technology, which has improved access to digital public services in recent times. With most countries adopting the e-ID means of identifying their citizens and ID holders, the federation of disparate and heterogeneous identity systems to create an interoperable ecosystem can be achieved globally. The motivation of this research is based on the premise that e-ID can strengthen the protection of identity credentials, promote social inclusion through access to digital services, and enhance the exchange of data between interoperating digital identity systems.

### Overview of identity management

The interactions between Service Providers and their users (or interchangeably ID holders) have become much more flexible due to the evolution of the Internet. In recent times, it has become possible to control the use of protected resources using several authorisation methods. Consequently, there is a need for the identification data of users or ID holders to be managed across disparate platforms. With the necessity to prove the validity of a user’s identity attributes such as name, address, age, gender, and other additional details during authentication processes, it is significant to have in place a trustworthy identity management system. According to Luong and Park (2023), an identity management system is useful for managing the identity attributes of users. To establish and prove an identity, such identity must first be created through a well-defined registration process. In a foundational identity management system, such a registration process involves the following (World Bank Group 2016):

1. Collection of identity attributes: Using biometric technologies and widespread enrolment, the national identity authority compiles each person’s distinct identity into electronic profiles.
2. Storage of identity attributes: The national identity authority maintains a database of data and creates an electronic registration of each person’s distinct identity in the nation. The authority also creates simple and uniform interfaces for this national identity registration.
3. Usage of identity attributes: By providing optional identification credentials, such as a national identity card, and identity verification services, the national identity authority encourages the usage of digital identities.

**Fig. 1:**
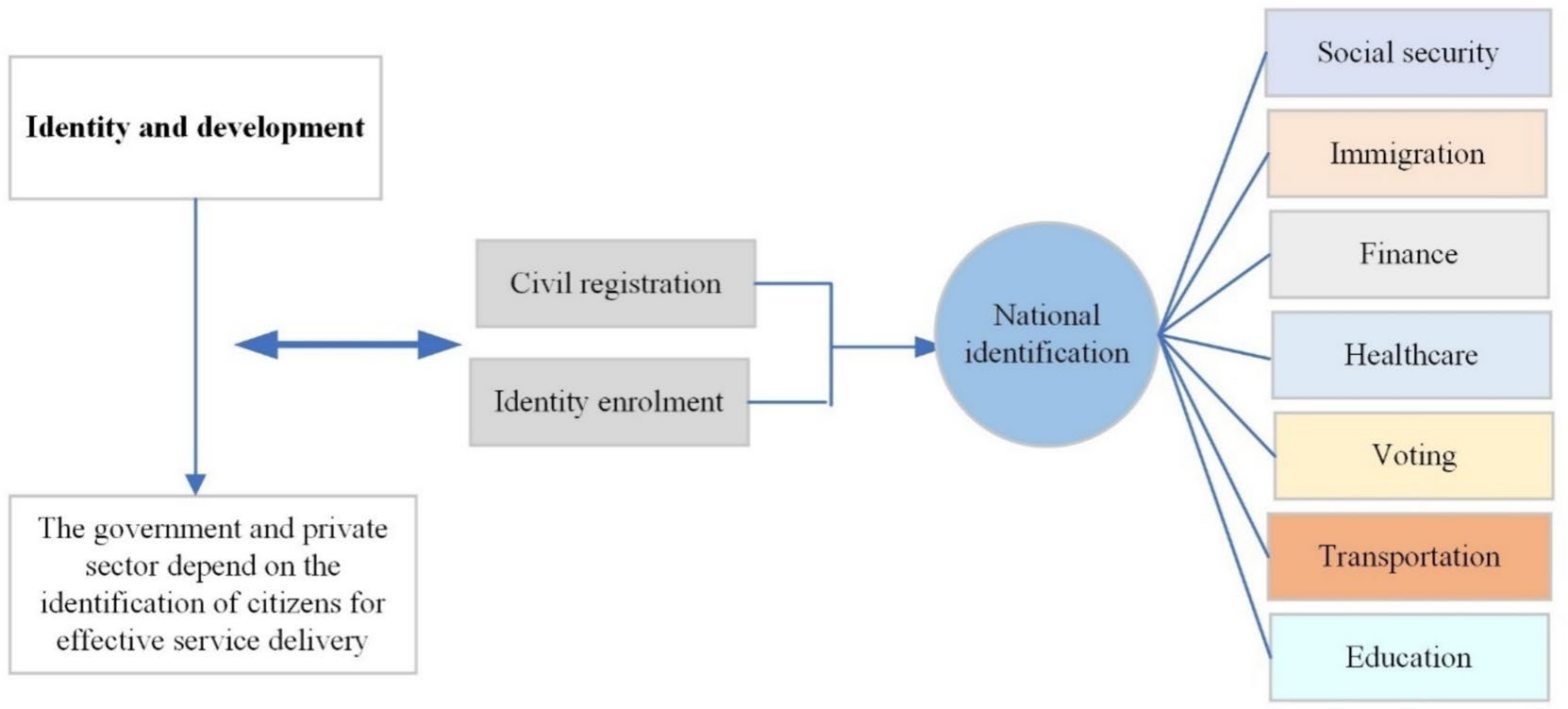
Identification as a key prerequisite for development

The processes of collecting, storing, and using identity attributes are useful for the verification and validation of ID holders whenever they request services from service providers. Relying parties also depend on these identity attributes to authenticate, authorise, and verify ID holders during transactions or access to a system (Kiourtis et al. 2023). Generally, identification is a key prerequisite for development as it provides the pivot for all categories of transactions and service delivery geared towards inclusive economic growth as summarised in Fig. 1.

### Trustworthiness in digital identity management

Building robust, secure, and reliable identity management systems is a crucial challenge worldwide. Fathalla et al. (2023) argued that trustworthiness in digital identity management ensures that identification data maintains its integrity, security, privacy, and confidentiality. The consequences of identity fraud or theft are discussed in Hummer and Rebovich (2023), Irvin-Erickson (2023), and Walters (2023). With the increasing rate of attacks on digital identities (Pöhn and Hommel 2023), privacy concerns are becoming more paramount. To this effect, the use of privacy-preserving technologies that do not reveal the real identities of ID holders is being considered (Luong and Park 2023; Tang et al. 2023; Yin et al. 2022).

Typically, a trustworthy system can be relied upon to authenticate and communicate identity attributes without which such information may not be trusted by identity and service providers including relying parties. When an identity system is compromised, it poses a direct threat to the digital identities of ID holders. Significantly, foundational identity systems must be trustworthy since they are part of a nation’s critical infrastructure that contributes to inclusive economic growth and e-government.

### Threats to digital identity systems

A digital identity is a unique representation of an ID holder or citizen in the context of a digital service or transaction. The evolution of digital identity systems has both merits and demerits for development. Gelb and Metz (2018) asserted that while ID systems can advance the SDGs of the UN by increasing opportunity, strengthening governmental capacity, improving accountability, and realising individual rights, they also have the potential to, at worst, encourage institutionalised discrimination, exclude vulnerable groups, and make it easier for personal data to be exploited. The recognition and verification of ID holders using invariable biometric features such as fingerprints and iris scans has changed the identification landscape, providing a platform for citizens and the government to interact including the implementation of developmental policies. As more citizens acquire identification credentials, their capacity to actively engage in the social, political, and economic life of their nation increases.

However, the handling of user credentials by most identification schemes has created an expanding threat landscape to digital identity systems. Issues of identity theft, impersonation attacks, denial of service, and distributed denial of service (DoS/DDoS) attacks in the management of digital identities have been discussed in recent studies (Sheik et al. 2021; Spagnoletti et al. 2018; Tewari and Mills 2021). To this effect, a thorough awareness of the threats related to identification schemes especially electronic identification (e-ID) systems is necessary for their security. According to Spagnoletti et al. (2018), in both the real and virtual worlds, identity theft is made easier by the trade of identity-related artifacts on the darknet.

Similarly, Tewari and Mills (2021) agree that although identities help citizens understand who they are through the control of information sharing, the digitisation of identity information and the development of digital identities bring with them complications. In this sense, both human and non-human entities may have the ability to add, remove, and/or alter identity information about others due to the extreme malleability of digital identities. This usually results in a loss of privacy and an increase in identity risks as well as privacy concerns. Moreover, the concept of digital identity as a legal identity adds another layer of complexity to the application of digital identities in many social and commercial contexts. Digital identity is challenging. Enforcing identity verification, particularly through digital means and remotely, presents a medium for an attacker to perform an impersonation attack (Grassi et al. 2017a).

Pöhn et al. (2023) evaluated the threats to self-sovereign identity (SSI), which enables user-centric and decentralised management of digital identities. With the electronic Identification, Authentication, and Trust Services (eIDAS) law by the European Union bringing the SSI technology into focus, the authors stated that it is critical to assess the threats to SSI since the storage and use of personally identifiable information (PII) is an integral component of identity management. They subjected the fundamental elements of the SSI architecture and its interrelationships to the STRIDE (Spoofing, Tampering, Repudiation, Information Disclosure, Denial of Service, and Elevation of Privilege) threat modelling methodology. Their findings show that various threat actors may attempt to read, edit, or remove data or communications on purpose or by accident. This is a significant concern for identity management based on the SSI paradigm.

### Challenges and opportunities of interoperability in the African identity ecosystem

The African identity ecosystem is fragmented with vendor-locked systems that are only accessible within the borders of each country (Gelb and Metz 2018). One of the challenges of interoperability in this context is the trust issues characterising the foundational identity systems of African nations (Domingo and Teevan 2022; Manda and Backhouse 2016). Trust is a key consideration to interoperability as government-to-citizen, government-to-government, and government-to-business interactions are performed over the Internet. Similarly, the increase in the use of vendor-neutral technologies for the verification and validation of ID holders at cross-border points mayintroduce more security and privacy concerns. This stems from the fact that implementing such a system may require the design of sociotechnical mechanisms to represent and strike a balance between all stakeholders in the ecosystem including their possibly competing interests (Calzati and van Loenen 2023). Moreover, cross-border digital trust requires a secure and reliable environment. Connecting identity systems can create more complex environments for conducting digital transactions and interactions across the continent (African Union 2020). Challenges in infrastructure, and disparities in social structures, norms, and behaviour, also affect the perception of privacy, security, and trust by stakeholders.

Other challenges include advancements in cutting-edge technology that change how personal data is gathered and analysed from various, unrelated sources and consent management for data sharing. According to the African Union (2020), the reluctance of various governments to invest in privacy-enhancing technologies, streamline security policies and legislation, and establish new acts for the protection of the privacy and confidentiality of identification data also add to the complexity of the identity ecosystem in the continent. Conversely, there are several opportunities for interoperability as it promotes vendor neutrality using common standards in an identity ecosystem. In the same sense, interoperability enforces data integrity by ensuring that each identity system provides a single source of truth for identification data and reduces identity fraud for e-government services (Domingo and Teevan 2022; The World Bank 2023b). With an interoperable continent-wide identity ecosystem, new markets, digital services, and applications are possible, thus enabling innovation and new use cases to widen the dimensions of e-government.

## Potential solutions

For the creation of effective, long-lasting, and usable identity ecosystems, interoperability is essential. Two major interoperability solutions in the identity ecosystem are discussed in this work. These include the Estonian X-Road and the electronic identification and trust services (eIDAS) of the European Union. These interoperability solutions were selected based on their widescale adoption and the fact that they are based on open-source technologies, which make their transactions provable by independent third parties(Engelbertz et al. 2018; Robles et al. 2019; Saputro et al. 2020; Schwalm and Alamillo-Domingo 2021). We note that there are other interoperability solutions such as secure identity across borders linked (STORK) (Ribeiro et al. 2018), Shibbolet (Kallela 2008), etc. However, these were not considered in this paper.

### X-Road

X-Road is a data exchange layer solution that implements the interoperability of information systems. It allows organisations to exchange data through a secure channel over the Internet. This solution is centrally managed but supports a distributed exchange layer that provides a standardised and secure channel for producing and consuming services (X-Road 2023a, b). X-Road enhances confidentiality, integrity, and interoperability between organisations that rely on it for data exchange (Bakhtina et al. 2022; Saputro et al. 2020). Jointly implemented by Estonia and Finland, it has been adopted by several countries and organisations (Robles et al. 2019; Saputro et al. 2020) and establishes online connections between service providers and data registries (such as the Population Register, Health Insurance Register, etc.). The citizens who use the X-Road system only give their information to the government once, and the public authority then stores and exchanges the information among itself via the X-Road system (Bhattarai et al. 2019).

By signing the messages with the X-Road member’s signature key and using a mutually authorised Transport Layer Security (TLS) channel, security servers guarantee the integrity and secrecy of the exchanged messages. By recording the exchanged messages and routinely timestamping the message logs, the signed communications’ long-term evidential value is protected. To obtain information on the validity of certificates and timestamp-signed messages, the security servers communicate with trust services. In terms of message exchange, the trust service calls are asynchronous (X-Road 2023c, d).

To exchange messages between a service client and a service provider, three protocols are used. These include (X-Road 2020):

1. X-Road message protocol: this protocol is used to establish communication between an information system and a security service within an organisation.
2. X-Road message transport protocol: this is a synchronous secure communication protocol that ensures privacy and reliability when sending messages between two security servers over the Internet.
3. OCSP response retrieval protocol: the protocol used in conjunction with the X-Road message transport protocol to create a secure channel of communication between the security servers of the service client and the service provider.

When an interested party such as an organisation joins an X-Road ecosystem, certificates issued by a reputable Certification Authority (CA) are used to verify the identification of each organisation and security server. Each security server serves as the technical entry point that manages access control on the organisation level during the data exchange process between registered X-Road members (European Commission 2023a; X-Road 2023a). While the identities are centrally managed, all data is transferred directly between a customer and a provider. According to the European Commission (2023a) and X-Road (2023e), the organisation and service level identifiers that X-Road maps to the physical network locations of the services provide the basis for message routing. No third parties have access to any of the data exchange evidence because it is all stored locally by the data exchange parties. The combination of timestamping and a digital signature ensures that data delivered via X-Road cannot be disputed (Blake Jackson et al. 2021).

### Electronic identification and trust services (eIDAS)

Electronic Identification and Trust Services (eIDAS) is a European Union’s framework to ensure that electronic transactions between businesses, citizens, and public agencies are safer and more efficient, regardless of the European country they take place. It was established by a European Regulation that was implemented in 2014. By introducing a common framework for eID and trust services, the eIDAS regulation makes it easier to supply business services across the EU. It encourages interoperability across the 28 EU nations, making certain that nations mutually recognise each other’s electronic identities and trust services across borders (Mocanu et al. 2019; Tsakalakis et al.^2019^).

The goal of electronic identification is to completely transform how customers engage with online services. The Member States of the EU may choose to identify citizens electronically. A small number of Member States have created national programs to provide their citizens with electronic identity (eID), with greatly diverse architectures (Lips et al. 2020; Tsakalakis et al.^2019^). National systems, therefore, vary not only in the volume of citizen data they process but also in the degree of data protection they provide to this data.

As claimed by Cuijpers and Schroers (2014) and Hölbl et al. (2023), businesses and customers can more easily access services or conduct commercial transactions by using electronic identity, or eID, to identify who they are (identification process) and demonstrate that they are who they claim to be (authentication process). Similarly, the regulation stipulates that it will be necessary for all EU nations to accept notified eID systems from other nations by September 2018. When conducting electronic transactions, especially those between firms and clients who are based in another EU country, trust services attempt to improve the trust of EU residents and enterprises (Sharif et al. 2022).

The trust services in eIDAS as discussed in ANSSI (2023) and European Commission(2023b) include the following:

1. Electronic Signature (eSignature): An electronic signature or eSignature is a person’s declaration of agreement with a document or set of data content in an electronic format. The same legal consequences as handwritten signatures apply to qualified eSignatures.
2. Electronic Seal (eSeal): the purpose of eSeal is comparable to that of the conventional business stamp. It can be used to ensure the authenticity and integrity of an electronic document.
3. Electronic Timestamp (eTimestamp): An eTimestamp ties an electronic document, such as a purchase order, to a certain moment in time, demonstrating that the document was present at that moment.
4. Website Authentication Certificates (WACs): these are electronic certificates that demonstrate to your clients the dependability and trustworthiness of your website. They make sure that the certificate holder is connected to the website. They aid in avoiding data phishing as well.
5. Electronic Registered Delivery Service (eDelivery): eDelivery allows the user to electronically send data. It offers evidence of the document’s transmission and delivery and safeguards your business from the possibility of loss, theft, destruction, or unauthorised revisions.

eIDAS has been exploited by both the public and private sectors in the EU. Some of the sectors that have benefited from the regulation include the financial services, online retail, transport, and professional services sectors. One of the largest potential beneficiaries of eID and trust services is the financial services industry due to the possibility of enormous commercial opportunities and enhanced cross-border services (Cuijpers and Schroers 2014; European Commission 2023b). To meet rising client demand for online services as well as stricter compliance requirements, the identification, authentication, and safeguarding of transactions in the financial services sector are becoming increasingly digitised.

### Challenges and limitations

#### X-Road

X-Road does not verify or validate the user’s identity data. However, it provides a secure layer for such data to be exchanged between subsystems of an information system, or in the case of federation, between federating identity ecosystems. Through federation, that is, joining together two X-Road ecosystems, X-Road offers built-in capability for cross-border data exchange. As if they were part of the same ecosystem, participants of the federated ecosystems can publish and consume services with one another. Multiple ecosystems can have federation connections made; however transitive federation relationships cannot be supported. If one ecosystem is not directly federated with another ecosystem, there is no federation link between them (X-Road 2023a).

There are a couple of challenges in the adoption and implementation of X-Road for trustworthy cross-border interoperability. These challenges are discussed in Freudenthal and Willemson (2017), Mikiver and Tupay (2023), Robles et al. (2019), and Saputro et al. (2020). They include the following:

##### Legal challenges

One of the legal challenges bothering the implementation of X-Road is the fact that interoperability is based on the signing of bilateral agreements, which are non-trivial and can result in several discrepancies in regulations and policies (Freudenthal and Willemson 2017; Krimmer et al. 2021; Robinson and Martin 2017). The Estonian X-Road, for instance, uses qualified signatures (or qualified digital seal), requiring that each X-Road member must have a qualified signature creation device. To acquire such a device to certify the related public keys is expensive and has lots of overhead. Conversely, the X-Road installation for Finland allows member states to choose their signature creation mechanism and message assurance level. This results in an inherent asymmetry in the trust levels of both the Estonian and Finnish X-Road instances in terms of message exchange (Freudenthal and Willemson 2017; Saputro et al. 2020).

Another important legal issue is the dispute resolution context for interoperating nations. This is important since dispute resolution for identity assertions, service requests, and fulfilments must occur within some jurisdiction. The added complexity to the actual implementation of cross-border digital services raises more concerns about the type of legal framework that is adequate for the wider federation context. It is also a non-trivial task to ensure that certification policies between federating systems are compatible since technical measures may not be able to establish all aspects of trust (Ansper et al. 2013; Freudenthal and Willemson 2017; Hoffmann and Solarte-Vasquez 2022).

##### Technical challenges

The service and trust level of the X-Road infrastructure and the messages sent over it are determined by several technological factors. These parameters must be equivalent across several federated instances to guarantee meaningful interoperability. The most significant difference between the Estonian and Finnish examples is in the global configuration and validity periods of the Online Certificate Status Protocol (OCSP) responses. Since the main goal of X-Road is to offer trustworthy data for decision-making, with the potential of holding the data source accountable if it can be shown that inaccurate data led to a poor decision, discrepancies in OCSP responses can mean that a compromised and revoked certificate can be accepted by members of an X-Road instance (Freudenthal and Willemson 2017).

The authorisation process for member organisations is another difficult component. Access control is exclusively addressed by X-Road infrastructure at the organisational level. The responsibility for end-user authorisation and access control within the information systems of the service client is performed by the service client. If these management procedures are insufficient, an X-Road member sharing its data sets runs the danger of a potential privacy breach brought on by a partner organisation’s negligent employee. It is highly challenging for an X-Road member to impose formal access control requirements on another company or to confirm that these criteria are met. When it comes to a foreign organisation, this issue is even more significant (Draheim et al. 2016; Paide et al. 2018; Freudenthal and Willemson 2017).

##### Root of trust

One of the fundamental ideas around which commerce and security are built is trust. A root of trust (RoT) is a source of confidence that is universally acknowledged (Grüner et al. 2020; Mocanu et al. 2019). The most widely used centralised RoT assumes that there is a centralised authority that the organisations have decided to trust. This type of authority is essential for ensuring the security of the identity management systems used by organisations. Organisations rely on the accreditation and calibre of the authority’s staff and the authority’s declaration of which entities can be trusted. The cryptography that is based on the foundation of trust is crucial to the security of identity management systems. Organisations using identity management systems based on the centralized RoT are hence vulnerable to a single point of failure (Bakhtina et al. 2022). 

Other challenges include:

1. There are different trust and assurance levels based on the implemented security architecture for verifying the integrity and authenticity of the data.
2. Incompatible certification authorities arise from different trust levels and legal systems of the trust service providers.
3. The fact that there are different legislations, technologies, and best practices between federating nations makes it very difficult to achieve a standardised set of well-implemented security measures.
4. The complexity of the current onboarding process with very few vendors having experience and knowledge of X-Road and its technologies. This leaves room for a plethora of consultations for any new joining member.

#### eIDAS

The goal of eIDAS is to provide legally secure electronic communications using a uniform framework and to increase public trust in electronic transactions within the EU (Gregušová et al. 2022; Lips et al. 2020, 2022). With eIDAS, each natural and/or legal person can make use of electronic identities to authenticate and verify themselves when required (Berbecaru et al. 2021; Pöhn et al. 2021). The provision of the mutual recognition and authentication of ID holders across the member states paves the way for an interoperable identity ecosystem. Citizens, businesses, and public administrators can use electronic identification and trust services including electronic signatures and seals, time stamping, registered electronic delivery, and web authentication to have access to electronic transactions (Kutyłowski and Błaśkiewicz 2023; Wagner et al. 2021). eIDAS is designed to achieve the following objectives:

1. Transparency and accountability
2. Security and trustworthiness
3. Technology neutrality
4. Standardisation

In eIDAS, there are challenges and compliance issues, which have been discussed in Hölbl et al. (2023), Muller-Torok and Bader (2022), Schwalm and Alamillo-Domingo (2021), and Schwalm et al. (2022). Some of these challenges include organisational independence, remote video identification, the use of electronic signatures in public administration, commercial access to the eIDAS network, and biometric authentication mechanisms. Technical issues with some of the mechanisms for providing security and authentication in eIDAS nodes include interpretation problems, different practices in member states, cooperation and collaboration barriers, and representation of legal persons.

## Towards trustworthy interoperability

**Fig. 2:**
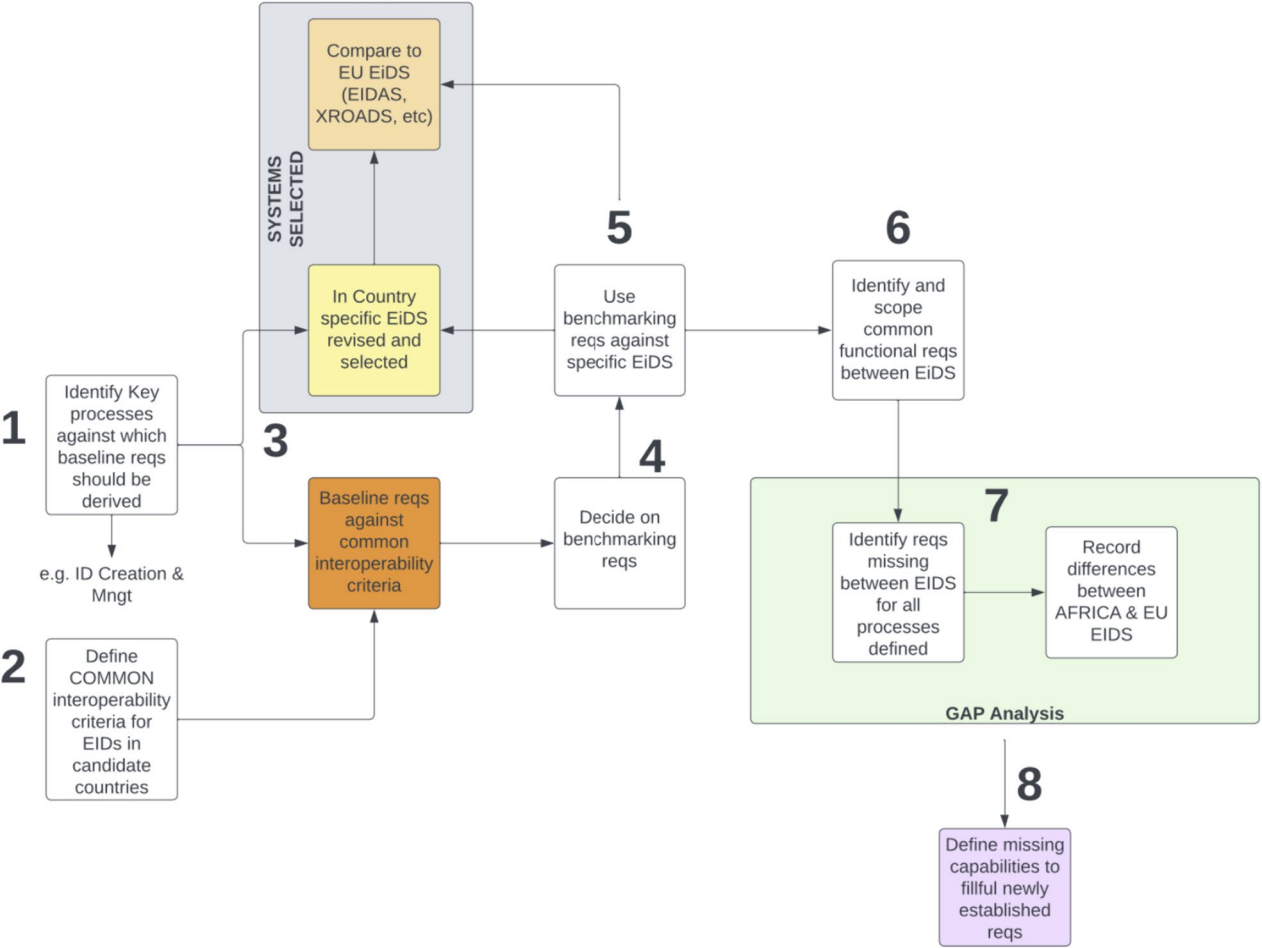
Proposed approach to scope requirements for interoperability

To underscore the importance of trustworthy interoperability in developing countries, an investigation and comparison of current interoperability solutions in the identity ecosystem was performed. This investigation identified the current limitations of existing solutions and provided the basis for our findings, which are relevant to achieving interoperability for foundational identity systems in developing nations. In investigating requirements for trustworthy interoperability in developing countries such as in Africa, the approach depicted in Fig. 2 is proposed.

As depicted in Fig. 2, to elicit requirements (reqs) for trustworthy interoperability in an identity ecosystem, it is relevant to identify the key processes against which baseline requirements should be derived. These key processes include identity creation and management, proof of identity, creating and issuing a credential, issuing a derived credential, managing the identity credential lifecycle, and granting access to an ID holder (Bertino and Takahashi 2010; Gelb and Metz 2018; Grassi et al. 2017a; Sullivan 2018; Windley 2005). The identification of the key identity management processes is significant in this context as it provides the basis for defining common interoperability criteria for eID schemes in the candidate countries seeking interoperability (Backhouse and Halperin 2009; Lentner and Parycek 2016). These criteria can then be compared with the baseline requirements. Notably, country-specific eID schemes should be compared with current interoperability solutions such as eIDAS and X-Road to identify common functionalities among eID schemes relative to the defined criteria. Building on this comparison, benchmarking requirements can be used against specific eID schemes to determine and scope common functional requirements useful for achieving interoperability. Furthermore, the missing requirements can be determined based on the defined key identity management processes by recording the differences between eIDschemes in Africa and the EU based on requirements gap analysis. Beyond this, missing capabilities can be defined to fulfill newly established requirements where the investigated eID schemes do not satisfy most of the baseline requirements for trustworthy interoperability.

### Comparison of X-Road and eIDAS as interoperability solutions

The comparison of X-Road and eIDAS is given in Table 1. This comparison is performed based on six (6) identity management processes, which include identity creation and management, proof of identity, creating and issuing identity credentials, issuing a derived credential, managing the identity credential lifecycle, granting access to an identity holder (Bertino and Takahashi 2010; Grassi et al. 2017a; Windley 2005), and other processes such as data exchange mechanism, security of identification data, and privacy of identification data.

**Table 1:** Comparison of X-Road and eIDAS

Process	X-Road	eIDAS
Identity creation and management	X-Road does not enforce any end-user identity scheme	Enforces identity schemes through the notification of national eID schemes
Proof of identity	End-user identities are not verified or validated	Verifies and validates end-user identities such as the eIDs of citizens or ID holders
Creating and issuing identity credentials	Issues credentials to X-Road members and security servers (SS). Does not issue credentials to citizens or ID holders	Creates and issues identity credentials to citizens or ID holders for mutual recognition of eIDs
Issuing a derived credential	Does not issue derived credentials to citizens	Issues derived credentials to citizens, which are part of the notified eID scheme
Managing the identity credential lifecycle	Manages identities of security servers, organisations and information systems (members) using a certificate authority (CA). The CA issues and revokes certificates to/of members	Manages identities of member states using a certification body designed by the European Commission to ensure that each member creates qualified electronic signatures and qualified electronic seals
Granting access to an identity holder	Access rights management is based on an authorisation framework using organisation and service level identifiers	Qualified web authentication certificates (QWAC) are used to authenticate the identification of the natural or legal persons to whom they have been issued, as well as the names of the relevant websites
Data exchange mechanism	Data is exchanged through message routing, which uses organisation and service level identifiers. These identifiers are mapped to the physical network locations of the services Non-repudiation of data is guaranteed through timestamping and digital signatures. Cross-border data exchange is achieved through the federation of X-Road ecosystems	Data exchange is achieved through an electronic registered delivery service (eDelivery), which allows the user to send data electronically eDelivery provides proof of sending and delivery of the data to curb the risk of loss, theft, damage, or unauthorised modifications of the data The content of the communication between eIDAS nodes is carried out with cryptographically secure Security Assertion Markup Language (SAML messages
Security of identification data	Authentication keys assigned to a Security Server (SS) are used to establish cryptographically secure communications with other SSs and TLS is used to secure messages transmitted over the public Internet	SAML is used to protect the confidentiality of the person identification data, the authenticity and integrity of the person identification data, and the secure identification of communication endpoints Transport Layer Security (TLS) is used to protect the communication between the client’s browser and server over the Internet e.g., connection via HTTP (HTTPS)
Privacy of identification data	Signing keys are assigned to the SS clients and used to sign the exchanged messages Messages are signed by the signing key of the organisations that send them Signatures are verified by the recipient of the messages A trusted certification authority issues certificates for the authentication and sign keys The security server verifies that certificates are issued by a trusted CA, have not expired, or revoked, and belong to the member organisation that is presenting them	Certificates for SAML signing and encryption of messages in the eIDAS network are exchanged through signed SAML metadata SAML request and response messages are signed SAML assertions are encrypted, and the encryption certificate is retrieved from the verified SAML metadata object of the requesting connector

With the approach provided in Fig. 2, stakeholders of identity systems can identify requirements at every stage of the identity management process. This is useful for building trustworthy identity ecosystems based on different patterns of federation (bilateral, multilateral, or arbitrary) to achieve the required level of interoperability that supports e-government across borders.

### Overcoming challenges and limitations

For governments and economies alike, enabling and bolstering reliable and secure identification in online transactions has become a constant problem (Gelb and Metz 2018). In today’s information era and the Internet, traditional identification through paper-based documentation or physical appearance rarely works (Cover 2015; Harbitz 2016; Sullivan 2018). Therefore, there is a pressing need for a digital version of paper-based identity that functions similarly to traditional identity cards or passports, capturing the unique qualities of an individual or organisation and setting them apart from others. According to Guenduez et al. (2023), this growth means that electronic identity, or e-ID, is now essential for both e-government and e-commerce to ensure safe online relationships including the interoperability of digital identity systems. e-ID serves two main functions: authentication and identification. These functions are characterised by uncertainties and risks, which can be resolved through trust.

Similarly, e-ID is an enabling factor for building an interoperable identity ecosystem when the relevant requirements are fulfilled since it enhances process integration for government applications, improves user convenience, and can allow a vast array of electronic services to be accessible to citizens from anywhere in the world at any time of day. It is anticipated that the enhanced accessibility and ease of e-ID-based services will have a favourable effect on interoperability. For cross-border data flow, e-ID can be used to provide e-services that transcend national boundaries and can assist in combating identity fraud both at the national and supranational levels. For example, e-ID is seen as a vital instrument in Europe for the delivery of e-services as well as the identification and tracking of international fraud (Atick 2016; Guenduez et al. 2023). Digital transformation, the establishment of a digital single market, and e-government are seen to require the capacity to engage and communicate with various governmental entities, even those located outside of one’s own country (Schmidt and Krimmer 2022; Torres et al. 2005). Therefore, overcoming the challenges and limitations of existing interoperability solutions can be considered in terms of trust relationships and assurance levels, trust frameworks, fulfilling requirements for interoperability, scalability analysis for service deployment, and resolving data encoding conflicts, which are discussed in the following sections.

#### Trust relationships

The implementation of an interoperable identity ecosystem requires the sharing of identity and user authentication credentials across the data exchange parties. One of the prerequisites for realising this data exchange between the interoperating identity systems is trust, which is crucial and can be accomplished by putting in place intricate contracts that outline shared policies, procedures, and obligations (Backhouse and Halperin 2009; Halperin and Backhouse 2012; Mbanaso et al. 2009; Tang et al. 2023). As a result, there is a trust link, where all participants are prepared to place their trust in one another’s claims. In an interoperable or federated domain, a set of service providers (SPs) can trust the IDs of users (citizens or ID holders) provided by identity providers (IdPs) from disparate SPs (Ansaroudi et al. 2023). In this sense, users can have access to the services provided by different SPs within the federated domain devoid of re-authentication. Federation protocols enable the interoperability of the identity ecosystems through a system of trust. Consequently, users can access services across the federation without being re-authenticated, thus enhancing service delivery.

The distributed and decentralised nature of services across an interoperable identity ecosystem means that the re-authentication of users who request services across different trust domains may lead to complexity in identity management since different services may need to store different credentials of users for every request. This creates a medium where the identity credentials of users are vulnerable to cyber threats. To this effect, interoperability will enhance the propagation of identity assertions to services in different trust domains to enhance the seamless communication of identity credentials through the establishment of trust. As opined by Kylau et al. (2009) and Hatakeyama et al. (2021), all interoperating entities or parties must rely on authentication assertions propagated within the federation by establishing well-defined trust relationships. These trust relationships are usually based on a set of contracts that outline the obligations and rights of each interoperating party as well as the policies they need to adhere to.

In establishing trust, three factors need to be considered. These include the reliance on the trustworthy party, the trustworthiness of the trusted party, and the cyber threats/risks associated with the failure of a trusted relationship (Guenduez et al. 2023; Kwame Adjei 2013). This concept implies that the risk that the parties to a trust relationship face in the event of a failure is directly correlated with the trust needs, or the procedures needed to develop trust. Here, the risk is defined as the product of two factors, which include the likelihood that an uncertain event will occur, and the consequences of that event (Halperin and Backhouse 2012; Van Staden and Bidwell 2023).

A user from identity ecosystem A can have direct access to a service provider in that ecosystem but indirect access to a service provider in identity ecosystem B. For an indirect access path, the mapping between the identifiers possessed by the same user would then need to be known by each service provider, if the client utilises distinct digital identities with various service domains. Indirect access is a secondary access path that does not require the client’s authentication credentials, whereas the principal access method of direct access requires the client’s digital identity and credentials for that domain. All that is needed for this indirect access method is for service providers to convey security assertions between them, such as Security Assertion Markup Language (SAML) assertions (Jøsang et al. 2005; Kylau et al. 2009). The trusted relationship that is established between IdPs and SPs is the fundamental component of interoperability through identity federation (El Haddouti and EL Kettani 2019). The digital identities of ID holders or citizens (users) are stored by an IdP, which then makes these identities—or parts of them—available to anRP who is prepared to rely on this information. The RP’s role is typically assumed by an SP in some cases. Therefore, users can verify themselves at a federated IdP and after successful authentication, they can rely on the assertion that the IdP issues (Carretero et al. 2018; Catuogno and Galdi 2014; Mohamed et al. 2021).

To understand the trust levels required for the interoperability of digital identity systems, we have identified the different levels of trust relationships in a federated domain. These comprise of:

1. User to Identity Provider ($User \mapsto IdP$)
2. User to Service Provider ($User \mapsto SP$)
3. Identity Provider to User ($IdP \mapsto User$)
4. Service Provider to User ($SP \mapsto User$)
5. Identity Provider to Service Provider ($IdP \mapsto SP$)
6. Service Provider to Service Provider ($SP \mapsto SP$)

These levels of trust relationships are summarised in Table 2.

**Table 2:** Levels of trust relationships for the interoperability of digital identity systems

Trust	Trust relationship					
	$User \mapsto IdP$	$User \mapsto SP$	$IdP \mapsto User$	($SP \mapsto User$)	$IdP \mapsto SP$	$SP \mapsto SP$
The IdP has put in place standard procedures for the registration/enrolment of users, the issuance of identity credentials, and the onboarding of users	✓		✓		✓	
The user has a personal authentication device that is tamper-resistant for authentication across different services	✓	✓				
The privacy of the user’s identity credentials must be protected by the IdP and SP	✓	✓	✓	✓		
From the user’s point of view, the IdP and SP have put in place adequate user registration processes and authentication methods	✓	✓				
The identity credentials of the user are accurate and tamper evident	✓	✓	✓			
An authenticated user can access a service in a federated domain based on identity assertions between SPs on behalf of the user		✓				✓
There is an accurate mapping of user identities between service providers		✓				✓
The SP strictly adheres to the acceptable policies for linking the user’s identity data from disparate SPs		✓		✓		

Although trust requirements can be difficult to implement, adhering to them helps to streamline the processes of managing the digital identities of users across different trust domains, which is relevant for achieving interoperability.

#### Levels of assurance for trust relationships

The realisation of trustworthiness in the interoperability of identity systems depends on the assurance levels of the processes and mechanisms used in creating, issuing, authenticating, proofing, and managing identity credentials. According to Grassi et al. (2017a) and the World Bank (2023), assurance levels rely on the strength of the process for proofing identities including the type of credentials and authentication mechanisms used at the time of a transaction. The identity proofing assurance level relies on the method of identification, the aggregated attributes of the user, and the accuracy of the attribute’s verification process (Grassi et al. 2017a). The method of identification is binary, that is in-person or remote while the process for verifying attributes can be based on deduplication or cross-checking. On the other hand, the authentication assurance level is based on the type of credentials, the number of authentication factors in use, and the strength of the cryptography used for protecting transactions from the likelihood of failures or risks (World Bank 2023).

The levels of assurance for digital identities based on the National Institute of Standards and Technology (NIST) framework (Grassi et al. 2017a, b; Temoshok et al. 2022; Ziegler et al. 2021) are shown in Table 3. Level 1 consists of identity assurance level 1 (IAL1), authentication assurance level 1 (AAL1), and federation assurance level 1 (FAL1). In level 2, we have identity assurance level 2 (IAL2), authentication assurance level 2 (AAL2), and federation assurance level 2 (FAL2) while at level 3, we have identity assurance level 3 (IAL3), authentication assurance level 3 (AAL3), and federation assurance level 3 (FAL3).

**Table 3:** Levels of assurance for digital identities

	Level 1 (low)	Level 2 (substantial)	Level 3 (high)
Identity Assurance Level (IAL)	Self-asserted identity, no proofing process	Remote or in-person identity proofing is required	Requires in-person or supervised remote identity proofing. Physical documentation must be examined to verify the identifying attributes of the ID holder
Authentication Assurance Level (AAL)	Single-factor authentication is required based on available authentication technologies (something you know, have, or are such as a PIN or password)	Requires two-factor authentication based on secure authentication protocols such as a token with a PIN or password	Requires at least two distinct categories of authentication factors and a hard cryptographic authenticator that is resistant to verifier impersonation
Federation Assurance Level (FAL)	Here, the relying party is permitted to receive a bearer assertion from an IdP. The assertion must be signed by the IdP with approved cryptography	FAL1 plus the requirement that the assertion is encrypted with approved cryptography such that only the relying party can decrypt it	FAL2 plus the requirement that the user presents proof of possession of a cryptographic key reference in the assertion

The use case determines which levels of assurance are chosen. Consequently, certain industries and transaction categories require higher levels of assurance than others. Because sensitive data is gathered and stored in financial and health systems, these sectors frequently demand a higher level of assurance than others. Similarly, the authentication architecture of the identity system should ideally be able to offer multiple levels of assurance suitable for various use cases (World Bank 2023). It is noteworthy to mention that there are many considerations when choosing the levels of assurance in trust relationships, as well as the identity verification procedures, credential types, and authentication techniques that make them possible. These criteria include the following:

1. The possibility of a failure, breach, or unauthorised release of sensitive information.
2. The risk that a failure or breach poses to people, organisations, programmes, and the public interest.
3. The convenience and inclusivity of the identity proofing and authentication processes; and the possibility that exclusion errors may arise due to higher levels of assurance.

In addition, levels of assurance are especially crucial for cross-border interoperability through mutual recognition and federation, as these processes require a digital identity system to meet a specific assurance level to be recognised for a particular purpose.

#### Trust framework

In an interoperable identity ecosystem, federation members must adhere to a set of rules and policies that underscore their mode of operation and communication. These rules and policies constitute the trust framework of the identity ecosystem and entail processes and procedures that provide assurance. In other words, a trust framework ensures that the bare minimum criteria for security, privacy, identity management, and interoperability are met (Grassi et al. 2017a; Temoshok and Abruzzi 2018; Temoshok et al. 2022). There are significant processes, which are an integral part of a trust framework. Temoshok and Abruzzi (2018) asserted that these processes include:

1. Performing identity management obligations.
2. Exchanging identity data.
3. Utilising data that has been shared.
4. Protecting and securing identity data.
5. Performing designated functions within the identity ecosystem.
6. Addressing legal and responsibility concerns.

To this effect, multilateral agreements that facilitate the trust and governance of an interoperable identity ecosystem are based on trust frameworks. Grassi et al. (2017a, b) and De Salve et al. (2023) stated that a trust framework is useful for creating trusted identities that will allow interoperability to function in the context of exchanging identity data between federating parties, that is, SPs, IdPs and RPs. In this work, we have identified the different components that should constitute a trust framework for trustworthy interoperability, especially in an African setting. These include ecosystem rules, technical standards, legal structure, compliance and enforceability, and compliance recognition and communication. These five (5) components define the mode of communication between interoperating parties and the expectations of each party to achieve trusted identities and transactions (Grassi et al. 2017a, b; Temoshok and Abruzzi 2018; Temoshok et al. 2022).

#### Fulfilling requirements for trustworthy interoperability

The cross-border sharing of identity assertions in an interoperable ecosystem leads to the emergence of new cyber threats and risks. To maintain the confidentiality, integrity, availability, and privacy of these shared assertions, there is a need for the requirements for building trust relationships to be established (Grüner et al. 2020; Jøsang et al. 2005; Kylau et al. 2009). In this work, we have identified a set of requirements, which are vital to achieving a trustworthy identity ecosystem. These requirements are summarised using Table 4.

**Table 4:** Trust requirements for trustworthy interoperability (system implies interoperable system)

Requirement	Description
Credential management	The system should provide a mechanism for citizens (ID holders) to enrol, proof, update, and delete identity information The IdP shall securely generate, update, and store the credential information The ID holder shall protect his/her identity credential and does not intentionally disclose it
Protocol translation	The system must translate the authentication and authorisation protocols in a bidirectional and lossless manner, for instance, with the use of SAML or OpenID Connect
Trust	The IdP must trust the SP to follow the established privacy policies concerning the non-disclosure of user data The SP must trust the IdP to follow the established privacy policies concerning the non-disclosure of usage statistics The IdP must trust the SP to follow the established policies and procedures concerning access control and delegated access When it comes to user registration, authentication, and identity mapping, the SP must trust the IdP to follow the established policies and processes The adherence of IdP Y to the established norms and processes for user registration, authentication, and identity mapping must be trusted by IdP X The SP must trust the IdP to attest to just the external SPs, which are federated with the IdP and are trusted to follow specific policies and procedures for delegated access. These policies and procedures are agreed upon between the IdP and the SP IdP Y must trust IdP X to uphold the mutually agreed-upon privacy regulations, which include keeping user data private The SP must trust IdP X to only grant authorisation for delegated authentication to federated IdPs that follow predetermined guidelines and protocols on user registration, authentication, and identity mapping IdP Y must trust IdP X to only provide permission for delegated assertion and user data consumption to federated SPs who comply with predetermined guidelines and protocols on user data non-disclosure The SP must trust IdP Y to only grant permission for delegated access to those federated IdPs who follow predetermined guidelines and protocols IdP Y must trust IdP X to only grant delegated access to federated SPs who follow predetermined guidelines and protocols
Authentication	The IdP shall implement secure and adequate user authentication mechanisms The SP shall manage the mapping of users correctly during authentication
Authorisation	The SP shall authorise access to protocol services by evaluating the assertions generated by the IdP The SP shall verify the user identity information based on an identity assertion from the IdP to authorise access to a service
Privacy	The system shall be developed to disclose only the essential identifying information of an ID holder to forestall identity theft The IdP, SP, and RP must protect the privacy of the user The IdP, SP, and RP must ensure that personally identifiable information will be kept confidential and used only to further the goals for which it was collected and authorised
Attribute management	The IdP must ensure that the delivered attributes of the ID holder are correct The IdP must revoke invalidated attributes promptly
Security	Communication between the interoperating systems through the browser of the ID holder shall be protected by Transport Layer Security (TLS) Communication over the authentication and authorisation protocols such as SAML or OpenID Connect must be cryptographically secure including the signing of all protocol communications and the encryption of all assertions The verifier must validate the signed object’s XML/JSON schema before confirming a signature. The decrypted content of a protocol message that contains encrypted portions must undergo further validation upon decryption For key storage, private cryptographic keys must be securely preserved The public keys that are used for identity assertion authentication must be kept in a way that prevents manipulation
Information assurance	Operators of interoperable identity ecosystems offering authentication must demonstrate that each system satisfies the requirements of standard ISO/IEC 27001 through certification, equivalent methods of assessment, or compliance with national laws Each system shall promptly apply security-critical upgrades within the ecosystem Vendors shall promptly provide any essential security upgrades

Although these requirements are not exhaustive, they provide the basis for building interoperable identity ecosystems. Furthermore, these requirements can be modified to account for the peculiarities of each operational interoperability framework.

#### Scalability analysis for service deployment

Performance and scalability analysis for the different lossless and bidirectional protocols for generating identity assertions are necessary for service deployment in an interoperable ecosystem. With the scalability of services limited by their underlying architecture and the increasing complexity and erratically increasing workload of services, the scalability of these protocols becomes critical. We will define scalability as a service’s capacity-boosting ability to use additional resources in the resource space, where resource space refers to the set of probable configurations of resources (Al-Said Ahmad and Andras2019; Antonov and Teplov 2016; Brataas et al. 2017). In this work, we will consider the following protocols:

1. SAML: With SAML, ID holders can have access to multiple services through SPs and IdPs. This technology offers the mechanism to authenticate a user only once and then share that authentication data with several applications (Almenárez et al. 2009; Ferdous and Poet 2013). However, it also introduces some performance and scalability issues. Some of these include increased network traffic, security vulnerabilities, and processing overhead. Optimising the performance and scalability of SAML is significant when deploying this protocol for use in regions with limited bandwidth such as in most developing countries with remote access to the Internet. Some of the ways that the performance and scalability of SAML can be optimised include the following:
  - Different bindings, or ways to transfer messages between parties, are supported by SAML. These include HTTP POST, HTTP Redirect, and Simple Object Access Protocol (SOAP). Every binding has merits and demeritson compatibility, security, and performance. For instance, HTTP Redirect is quick and easy to use, but it can reveal sensitive information in the URL and has a size limit. Larger messages can be handled by HTTP POST, which is more secure, but it necessitates more processing and user involvement. Although SOAP increases network overhead and complexity, it is dependable and compatible. To optimise SAML, it is significant to choose the binding that best fits the use case, requirements, and limitations of the system in focus (Bianco et al. 2007; Nordbotten 2009).
  - Components, including assertions, signatures, extensions, metadata, and encryption can be included in SAML messages. These components may have an impact on the message’s size, which can affect the scalability and performance of this protocol. Larger messages, for instance, may surpass the capacity of specific bindings or platforms and require more bandwidth in addition to taking longer to transmit and process. As a result, the message size should be reduced by eliminating redundant components, compressing them, utilising references rather than data embedding, and dividing bulky messages into smaller ones (Carretero et al. 2018; Chadwick et al. 2014).
  - Statements that include details about a user, including their identity, attributes, and authentication status, are known as SAML assertions. The IdP issues assertions, which are then used by the SPs to authorise user access. Performance and scalability costs can arise from the generation and validation of assertions, particularly when complicated logic, many sources, or cryptographic procedures are involved. As a result, if an assertion is still true and pertinent, it should be cached and utilised again. This can accelerate the authenticate process, lessen the strain on the IdP, and enhance customer satisfaction (Mainka et al. 2016).
  - For SAML to be efficient, both the IdP and the SP must be accessible and dependable. Therefore, load balancing and failover methods must be put in place. To manage SAML requests and responses, for instance, numerous servers, clusters, or cloud services can be utilised. In the event of a failure, it should be possible to migrate to other servers or providers (Schwartz et al. 2018).
  - Sensitive data, including the credentials of ID holders, attributes, and session tokens, are exchanged between the IdP and the SP with SAML. The security and integrity of the process may be compromised if these data are intercepted, altered, or falsified by malicious users. Therefore, to safeguard the confidentiality, authenticity, and non-repudiation of the SAML messages, there is a need to secure the communication channels between the parties using encryption, signatures, certificates, and other techniques (Carretero et al. 2018; Chadwick et al. 2014; Schwartz et al. 2018).
  - SAML may be difficult to understand and prone to mistakes, particularly when several platforms, parties, and standards are involved. It can be challenging to assess and enhance SAML’s performance and scalability as well as to find and fix the core cause of issues with the authentication process. As a result, it is relevant to track and examine the SAML messages, events, and results while monitoring and debugging the authentication process utilizing tools, logs, audits, and metrics. This can be useful in finding and resolving problems, streamlining the authentication process, and improving the effectiveness and quality of authentication (Nordbotten 2009; Schwartz et al. 2018).
2. OpenID Connect: this is an interoperable authentication protocol, which is developed based on the OAuth 2.0 framework of specifications (Alrodhan and Alqarni 2017; Mainka et al. 2017; Naik and Jenkins 2017). OpenID Connect streamlines the process of obtaining user profile data in a REST-like and interoperable manner, as well as user identity verification based on authentication carried out by an authorisation server. It provides users with a universal identity and allows them to sign into many relying parties (or RPs) (Alrodhan and Alqarni 2017; Zhang et al. 2017). Because OpenID enables single sign-on, it eliminates the need for unique passwords and signatures on every OpenID-compatible website. However, there are multiple security flaws in the OpenID verification process. According to Alrodhan and Alqarni (2017), phishing attacks are one major risk inherent in the OpenID authentication process. Additionally, flaws in the web single sign-on architecture allow attackers to execute deception attacks (Mainka et al. 2017).Some of the scalability issues with OpenID Connect include:
  - The various protocol flows that OpenID Connect offers result in notable variations in the messages that are sent back and forth between the entities. Three distinct major flows exist in OpenID Connect: coding, implicit, and hybrid. Different parameters and messages are expected by each flow. This raises the level of analysis that is required. The end-user verifies the id_token in the implicit flow on its own. For example, the SP transmits JavaScript code to the end user’s browser as a result. In the other flows, server code is used to validate the id_token on the SP. As a result, several implementations on a single SP require independent validation.
  - Mobile applications, native applications and web applications are the three SP kinds that OpenID Connect defines. Every category needs a different set of messages and flows. Furthermore, distinct security considerations need to be taken for every SP capability. For instance, when utilising OpenID Connect on a web application, the connection between the web application and the IdP is hidden, making it impossible for an attacker to view the full exchange. On the other hand, the attacker has complete control over the mobile device, making it simple to monitor and even alter the communication between an installed application and the IdP, for instance, by using an HTTP proxy.

#### Resolving data encoding conflicts

One of the challenges to the interoperability of digital identity systems in developing countries is the issue of data encoding conflicts. To address this challenge, it is important to consider the following:

1. Data encoding conflicts
  - Standardisation: For an interoperable identity ecosystem, developing countries must adopt industry-standard encoding formats such as UTF-8 for text data and standardised binary formats for non-text data, as recommended by the World Wide Web Consortium (W3C 2023). This will ensure consistency and compatibility across different identity systems.
  - Validation and Transformation: Interoperating systems must implement robust validation mechanisms to detect encoding issues early (Lee et al. 2021). There is the need to also develop transformation tools to convert data into the required format seamlessly, thereby minimising encoding conflicts (Rahrooh et al. 2024; Reis and Housley 2022).
  - Documentation and Training: For a seamless interoperable ecosystem, providing comprehensive documentation and training on supported encoding formats and handling various data types is crucial for developers and users (Sun et al. 2020).
2. Conflicts from underlying features
  - Use of Modular Architecture: It is important to design the digital identity platform with a modular architecture to allow different features to operate independently, which reduces the risk of conflicts (Mesa et al. 2020). This approach enables easier updates and integration with other systems.
  - Conflict Resolution Mechanisms: We posit that establishing clear protocols for conflict resolution, such as priority rules and arbitration processes, to manage feature interactions will engender interoperability (Crespo et al. 2007). This will include conflict detection algorithms and fallback procedures to ensure system stability.
  - Testing and Continuous Integration: There is a need to implement extensive testing procedures, including automated and manual tests, to identify and address conflicts early in the development cycle of an interoperable identity system (Jaffar-ur Rehman et al. 2007). Continuous integration practices will help in maintaining system integrity as new features are added.

By focusing on these strategies, this research aims to enhance the interoperability of digital identity systems and ensure a smooth, conflict-free user experience, particularly in developing countries where diverse technological ecosystems can pose additional challenges.

## Discussion of findings

The limitations of the current interoperability solutions, that is, eIDAS and X-Road show that there is a plethora of issues and complexities, which are yet to be addressed to achieve interoperability at the foundational level of identity management. Country-specific legislation and vendor lock-in in developing countries such as sub-Saharan Africa add to these complexities.

From the findings, the main limitations of the interoperability solutions examined can be summarised as follows:

1. Each registry implements its interfaces independently based on the use of a proprietary protocol equivalent to the technology in use. This results in the implementation of new interfaces, sometimes from scratch for new or evolving services.
2. Depending on the security architecture in place for confirming the authenticity and integrity of the data, there are various levels of trust.
3. Incompatible trust levels and legal frameworks among trust service providers give birth to incompatible certifying authorities.
4. Interoperability is based on the signing of bilateral agreements, which are non-trivial and can result in several discrepancies in regulations and policies.
5. In eIDAS, there is no provision that the network must be accessible to private entities and as such may build inter-government competition on trust services.
6. Also, in eIDAS, there is differentiation in notified eID schemes and authentication mechanisms leading to re-identification for public services, healthcare, or financial transactions.

These limitations imply that the interoperability of identity systems requires open standards with strong legal, regulatory, and governance structures. Also, there must be mechanisms to mitigate risks to the security and privacy of identification data including consent considerations for data use or sharing by the ID holder as outlined in Alamillo et al. (2023) and Srinivas et al. (2019). From the findings, developing countries must also make provisions for a single, consolidated, and standardised view of civil registrations and identification data that constitute a single source of truth. This will enhance the onboarding of citizens and create a robust verification and validation process that does not require several levels of authentication that may increase the overhead of the identity system.

Additionally, findings showed that the standardisation of the structure and attributes of identity data such as name, date of birth, email, and several other relevant attributes to conform with the W3C recommendation should be a key consideration for interoperability. Enforcing the unicity and singularity of identification data will also ensure that ID holders do not have multiple identities that can hamper interoperability. Similarly, to enable the secure exchange of data or identity assertions, it was also found that developing countries must establish trust relationships through federation protocols that can foster interoperability.

Interoperability portrays tremendous benefits to e-government. We found that interoperability widens the dimensions of e-government in cross-border identity management and data services. It also helps to provide open and accessible digital public services including systems and processes that allow people to move freely within developing countries while also utilising public services outside their country of origin. Interoperability also helps to create sustainability, and economies of scale as demonstrated by X-Road and eIDAS (Hoffmann and Solarte-Vasquez 2022; McBride et al. 2019; Schmidt and Krimmer 2022).

We found that while X-Road provides trustworthy data exchange using security servers that allow its members to communicate directly, it does not perform the verification and validation of the identification data that is part of the data exchange. There are also concerns about the limited amount of notified eID schemes under eIDAS, which builds on the limited scope of the eID schemes and the lack of relevant public services. These concerns underpin the need for the review of these interoperability solutions to underscore the notion of cross-border verification and validation of identification data for seamless data exchange in an African setting.

In a cross-border use case, where a foundational identity system ${NIDS}_{x}$ communicates with another system, say, in a federated identity ecosystem ${Fed}_{ID}$, or where an ID holder ${U}_{ID}$ requests for a service from the latter, then the processes of verifying and validating the identity of ${U}_{ID}$ should be an integral component of an interoperable identity system unlike in X-Road where such processes are the functions of the service provider/consumer. Verification and validation ensure that the claimed identity is true and belongs to the claimant at the time of the request and throughout service delivery. To achieve this, there is the need to have a trustworthy link that considers the representation of the data, semantics, binding, and the security and privacy of the identification data of ${U}_{ID}$. The relevance of the trust link at the interoperable layer is to ensure that the identity data as well as the requested resource or service maintains its integrity, security, privacy, and confidentiality throughout data exchange.

We argue that the representation of the identification data must be data format agnostic as obtainable in X-Road using simple object access protocol (SOAP) and representational state transfer (REST) (Halili and Ramadani 2018; Krimmer et al. 2021; Priisalu and Ottis 2017). Also, the representation of credentials on the Web should be in a way that is machine-verifiable, private, and cryptographically secure. The semantic interpretation of ID credentials must be unambiguous. That is, verifying credentials and presentations and cryptographically securing them both require predictable, bi-directional, and lossless processes. To be processed in an interoperable manner, any verification of a credential or presentation must be deterministic. The resulting credential or presentation must be semantically and syntactically equivalent to the original construct (Sedlmeir et al. 2021; W3C Recommendation 2022). Likewise, each verified credential of ${U}_{ID}$ must be bound to its identity to a given level of assurance. This establishes an unbreakable link between the subject (${U}_{ID}$) and the credentials to enforce identity disambiguation. Binding is relevant for the verification and validation of the claimed identity at cross-border entry/exit points.

Finally, the security and privacy of the identification data are significant as they form the integral component of the required trustworthiness for interoperability. There are several approaches for implementing the security and privacy of identification data such as the use of Transport Layer Security (TLS), Security Assertion Markup Language (SAML), authentication keys, etc. (for security), secure computation mechanisms, trusted third party, differential privacy, etc. (for privacy) (Grassi et al. 2017a; Kaaniche et al. 2020). We posit that the use of secure computation mechanisms, data minimisation, and differential privacy in a cross-border context can fulfil the required privacy requirements due to the multifaceted risks associated with the exchange of data between interoperating entities in the African identity ecosystem.

## Conclusion

Trustworthy interoperability is a key enabler for identity management and widens the dimensions of e-government in developing countries. This research pointed to the challenges, opportunities, and requirements of interoperability and highlighted the limitations of the examined interoperability solutions such as the Estonian X-Road and the eIDAS of the European Union. We found that there are incompatible trust levels and legal frameworks within the African identity ecosystem, which can affect the seamless communication of identity assertions in an interoperable context. With various levels of trust for confirming the authenticity and integrity of data, this incompatibility could mean that several discrepancies may arise in regulations and policies among interoperating systems especially where there are no existing strong legal, regulatory, and governance structures. With the fragmentation of the identity ecosystem in Africa, our findings show that developing countries should consolidate and standardise the civil registration and identification data into a single source of truth to eliminate multiple identities. Establishing this unicity and singularity of identity data is significant for enforcing identity disambiguation and achieving trustworthy interoperability. Similarly, it is noteworthy that mechanisms are established to mitigate imminent risks to the security and privacy of identity data.

To achieve seamless communication of identification data, we found that developing countries must establish trust relationships through federation protocols and define the assurance levels of the processes and mechanisms used in creating, issuing, authenticating, proofing, and managing identity credentials. In the same sense, interoperating systems must establish (and adhere to) a trust framework that outlines the set of rules and policies that underline their mode of operation and communication. Furthermore, the requirements for building trust relationships should be determined based on our proposed approach. There is also the need to perform scalability analysis for the different lossless and bidirectional protocols for generating identity assertions, which are essential for service deployment in an interoperable perspective. These findings add to the body of literature on trustworthy interoperability of identity systems with the aim of supporting the development of cross-border e-governance within the African identity ecosystem.

## Data Availability

The manuscript has no associated data.
